# Sociocultural and Economic Dimensions of Rift Valley Fever

**DOI:** 10.4269/ajtmh.14-0363

**Published:** 2015-04-01

**Authors:** Geoffrey Otieno Muga, Washington Onyango-Ouma, Rosemary Sang, Hippolyte Affognon

**Affiliations:** Institute of Anthropology, Gender and African Studies, University of Nairobi, Nairobi, Kenya; International Centre for Insect Physiology and Ecology, Nairobi, Kenya

## Abstract

Health researchers have advocated for a cross-disciplinary approach to the study and prevention of infectious zoonotic diseases, such as Rift Valley Fever. It is believed that this approach can help bring out the social determinants and effects of the zoonotic diseases for the design of appropriate interventions and public health policy. A comprehensive literature review using a systematic search strategy was undertaken to explore the sociocultural and economic factors that influence the transmission and spread of Rift Valley Fever. Although the findings reveal a paucity of social research on Rift Valley Fever, they suggest that livestock sacrificial rituals, food preparation and consumption practices, gender roles, and inadequate resource base for public institutions are the key factors that influence the transmission. It is concluded that there is need for cross-disciplinary studies to increase the understanding of Rift Valley Fever and facilitate appropriate and timely response and mitigation measures.

## Introduction

Rift Valley Fever (RVF) is an acute viral zoonosis that affects cattle, sheep, and goats but also, people and wildlife.^1^ RVF is, primarily, transmitted by the *Aedes* mosquito and breaks out during unusually severe rainfall. According to the World Health Organization (WHO),^2^ RVF is one of the emerging infectious diseases that mainly affects the poor and marginalized populations that lack access to health services and are readily ignored. As a result, these populations are subjected to a cycle of ill health and poverty that aggravates their burden of infectious diseases. The majority of human infections takes the form of mild fever, but a small percentage (< 1%) leads to more severe manifestations, including fatal hemorrhagic disease.^3^,^4^

Humans usually get RVF through bites from infected mosquitoes. RVF virus (RVFV) infection can also occur in humans if they are exposed to the blood, body fluids, or tissues of infected animals. Direct exposure to fluids of infected animals can occur during slaughter or through veterinary and obstetric procedures. Hence, the risk of infection is greatest when slaughtering in the context of traditional sacrificial practices, on which occasions aerosols of infected blood are likely to be generated.^2^,^3^ This is the major reason that outbreak of RVF is commonly associated with people whose livelihoods revolve around livestock rearing. Neutralizing antibodies to RVFV have also been shown in wildlife in Kenya, including African buffalo, black rhino, lesser kudu, impala, African elephant, kongoni, and waterbuck. This raises the possibility that wildlife may be reservoirs for the virus during interepidemic periods and play a role in amplifying the virus during epizootics.^4^–^7^

The virus that causes RVF was first isolated in 1930 during an investigation into an epizootic among sheep on a Naivasha farm in the Rift Valley of Kenya.^1^–^9^ Since 1930 when the first cases were diagnosed, mitigation measures have tended to emphasize the veterinary and health perspectives, with a lot of attention paid to monitoring and reporting of cases and death incidences to the veterinary and health authorities. However, the sociocultural and economic contexts within which RVF occurs remain a neglected research province.^9^,^10^ How, for example, do the norms, ideologies, and practices of pastoralists underpin the outbreak, spread, and effects of RVF on herds and human health? These questions resonate with the current epidemiological discourse calling for an evolution of theory and practice that would move the field from a focus on proximate, independent risk factors toward new paradigms of distal, interconnected determinants of disease risk.^11^

The importance of social factors as drivers of disease occurrence and spread has been well-established.^12^ A key difference identified by some social epidemiologists in their frameworks for understanding disease processes has been the focus on social conditions that promote or harm health rather than specific health outcomes.^13^,^14^ It has been argued that such inclusiveness is required by the fact that all diseases can be considered to be products of both biological and social processes. It is clear that social, political, behavioral, and environmental factors shape the emergence and re-emergence of infectious diseases.^15^,^16^ Farmer^16^ discussed the role of social inequalities in the recent emergence of infectious diseases, such as Ebola, Acquired Immune Deficiency Syndrome (AIDS), and tuberculosis. For these reasons, Farmer,^16^ Bates and others,^17^ and Ali^18^ have advocated a social determinants approach to the study and prevention of infectious diseases at the population level.

This article explores the sociocultural and economic factors that influence the transmission and spread of RVF disease. We undertook a systematic and comprehensive review of literature to identify and analyze the sociocultural and economic dimensions of the disease that have been studied and documented.

### The RVF hotspots.

After the discovery of RVF (RVFV) in 1930 among sheep on a Naivasha farm in the Rift Valley of Kenya,^19^ extensive outbreaks were not reported until 1951, when an estimated 20,000 persons were infected during an epizootic of cattle and sheep in South Africa.^20^ In 1950 and 1951, a main epizootic occurred in Kenya, resulting in 5,000,000 sheep abortions and 100,000 sheep deaths.^7^ RVF later occurred in epizootic form in northeastern Kenya in the years 1961 and 1962. Since then, outbreaks have occurred in a number of countries, notably Egypt in 1971–1978; Egypt and Senegal in 1993; Kenya, Somalia, and Tanzania in 1997 and 1998; and Saudi Arabia and Yemen in 2000 and 2001.^4^–^7^ It is estimated that approximately 27,500 human infections occurred in Garissa, northeastern Kenya in 1997 and 1998, the largest ever recorded outbreak of RVF in east Africa.^7^ A multivariate analysis revealed that contact with sheep and body fluids and sheltering livestock in one's house were significantly associated with infection and suggested that public education during epizootics has the potential to reduce human illnesses and deaths associated with RVF outbreaks.

In east Africa, RVF has been reported in arid and semiarid areas in the form of sudden and dramatic epidemics at intervals of approximately 10 years associated with widespread flooding and the resultant swarms of mosquitoes.^5^ In 1997–1998 and 2006–2007, massive outbreaks of RVF occurred in east Africa, and both were associated with El Nino events.^6^,^7^ When the 2006–2007 outbreak subsided, more than 1,000 people had been diagnosed with RVF, and more than 300 people had been confirmed to have died of the disease.^8^

In Kenya, outbreaks have occurred in the northeast, where a nomadic way of life is the predominant social order and livestock rearing is pivotal to livelihoods. The last major outbreak of RVF experienced in Kenya occurred in 2006, with the last case being confirmed in June of 2007. This together with the 1996/1997 outbreak were the most notable in terms of public health and socioeconomic impact, thereby attracting unprecedented research interest.^21^ The Kenyan site covers areas of Garissa, Ijara, and Lamu located between the Tana River and the boundary to Somalia. This area is a very flat floodplain stretching from Garissa in the southeast direction toward the coast, with little topography, perennial river valleys, and gentle local elevations. The area is semiarid with low undulating plains that have low-lying altitude ranging between 0 and 90 m above sea level and vegetation cover of shrubs and acacia bushes.

This semiarid area has two rainy seasons per year: the long rains from March to April and the short rains from October to December. Typical annual rainfall averages between 300 and 500 mm,^1^ although there is high interannual variability. The rainfall is unreliable, with some short periodic torrential downpours. The temperatures are often high, ranging from 20°C to 38°C.

### Population and livelihood.

An outbreak of RVF is commonly associated with people whose livelihoods revolve around livestock rearing, a common practice in the above-mentioned hotspots, especially northeastern Kenya, where the most recent outbreak occurred in 2006 and 2007. The inhabitants of the northeastern Kenya region are predominantly Somali pastoralists practicing livestock keeping as the main economic activity. Although a number of settled towns are dispersed throughout the region, the rural population is principally composed of nomadic people. About 90% of the population is directly dependent on livestock for daily population nourishment and as an income resource.^21^ During the last outbreak (2006 and 2007), a ban on livestock trade and an imposition of a quarantine resulted in severe economic losses that run to greater than US $9.3 million.^21^ The livelihood is constrained by the high prevalence of diseases, such as RVF, poor marketing of livestock, and frequent droughts. Pastoralism, however, remains the most viable economic activity in the region.

## Review Design and Method

Two different search strategies were used for this review. First, we conducted a literature search on the PubMed database using the terms “Rift Valley Fever,” “Rift Valley Fever in Kenya” (because Kenya experienced two consecutive epizootics in 1996/1997 and 2006/2007), “social determinants of Rift Valley Fever,” and “sociocultural and economic factors of Rift Valley Fever.” Second, we collected unpublished literature on RVF in Kenya. These data were mainly reports obtained from research institutions and government departments and media materials. The unpublished literature was deemed important to support the first search strategy and also, reveal the level of knowledge management of the zoonotic disease. A rigorous thematic content analysis was used to identify, document, and analyze the sociocultural and economic dimensions of RVF disease.

## Limitation

We recognize that literature database searches alone may not find every relevant citation. However, our aim was to put together a body of literature that can show the usually ignored argument that RVF, just like other infectious and zoonotic diseases, has sociocultural and economic dimensions worth integrating in response and mitigation measures.^9^,^10^ These dimensions are also useful for consideration in designing public health policies and decision-making during interventions.

## Findings and Discussion

We conducted a search of the PubMed database to identify literature using the term “Rift Valley Fever,” which yielded a total return of 1,321 citations. Also, an attempt was made to focus the search by using the term “Rift Valley Fever in Kenya” given that the country most recently experienced two major outbreaks (1996/1997 and 2006/2007), and this yielded a return of 117 citations. A search using the terms “social determinants of Rift Valley Fever” and “sociocultural dimensions of Rift Valley Fever” yielded zero return. Of all of the citations accessed, 33 peer-reviewed articles were purposively selected for review on the basis of having some materials on sociocultural and economic dimensions of RVF.^22^–^56^ Additionally, seven unpublished reports from research institutions and Kenya Government Departments and international conference papers were also reviewed as shown in Table 1.

A detailed review and analysis of the literature revealed that almost all of the materials accessed were biomedical-oriented, focusing mainly on veterinary and health perspectives of RVF. The sociocultural constructs of the disease are not comprehensively documented and are mentioned only in limited instances in the literature. The review reveals a paucity of social research on RVF disease.

## Sociocultural and Economic Dimensions of RVF: Sociocultural Dimensions

The sociocultural dimensions are described within the three-tier framework proposed by Rushton and Yrjo-Koskinen,^22^ namely the context in which diseases circulate: the value chain and the rules in which people in the value chain operate, institutional environment and the response of the people concerned, and human behavior.

The framework by Rushton and Yrjo-Koskinen^22^ emphasizes that the livestock food value chain has stages where pathogens can be maintained, spread in both directions, and introduced from external sources. These stages together with their pathogen conduits are feed inputs (feeds), production system (animals), transport (animals), abattoir (carcass), processing and marketing (meat), preparation (meat), and consumer (meat). Rushton and Yrjo-Koskinen^22^ further hold that the livestock food system provides food, moves money, and generates employment and that the people in the chains are geographically dispersed, a fact that presents a great likelihood of moral hazard.

A study of the livestock value chain for animal health measures is important in many ways, because a value chain is no different to a biological organism.^22^ It survives to support the livelihoods of the people who work in it and feed the people who are its consumers. If a disease is put into a value chain, the people within will react and modify their behavior. In turn, the people's actions will affect how the chain functions and operates. Strong chains will manage and internalize disease risks. Understanding how the livestock value chains modify and manage disease allows us to help see how our interventions can help a chain to recover as fast as possible. The rapid healing of a chain is vital to ensure that people who depend on the chain for income and food are affected as little as possible.^22^

### The livestock value chain analysis and RVF disease: human behavior, livestock production, and institutional environment.

#### Human behavior/responses.

Rushton and Yrjo-Koskinen^22^ reiterate that people are no longer just the people affected by a disease. They suffer lost income, ill health, and even death. People's actions within the value chain dictate how a disease enters a society, how it spreads, and how it is controlled; hence, risk management with a people-centered approach is needed. With a people-centered approach, it becomes critical to understand people's behavior. Some of this will be dictated by economic incentives, institutional environment rules (official and informal) and their enforcement, and social, cultural, and psychological factors.

The nature of rules, practices, human behavior, and perceptions about risks that define the social context within which a disease breaks out, spreads, and is controlled has prominently featured in ecological analyses as confounding elements of operations research on diseases. The review revealed the following behavioral practices as drivers of RVF transmission and spread in animals and humans.

#### Livestock sacrifice rituals and RVF transmission.

A study on RVF by Davies^23^ gives a clear understanding of the role of sociocultural practices in transmitting and spreading RVF. The experience of haj in Mecca has shown that one of the principal practices that exposes communities to infection with RVFV is the ritual sacrifice of rams by halal. This takes place in the midst of a huge number of people, thereby exposing the crowd to the risk of infection with RVFV in the event that the animal is infected at the time of slaughtering.

Davies^23^ has provided an account of how the supply of rams to meet this animal demand for the haj festivals through trade from all pastoralists' areas in east and northeast Africa and the Horn of Africa to Saudi Arabia exposes the people involved to the risk of infection with RVFV. Davies^23^ particularly notes that the animals have tended to originate from the semiarid pastoral zones of northeastern Kenya, Somalia, southeast Ethiopia, and western Sudan. During such festivals, Davies^23^ reports that the proximity of high densities of people and the large numbers of animals being slaughtered by halal present a hazard. If the blood is infected with zoonotic pathogens, these may be disseminated to the population during the halal ceremonies by droplets or aerosols or through the skin by wound contamination.^23^

In the Kenyan case, it has not been established whether the incidences of RVF outbreak in the northeastern parts of the country have ever coincided with similar practices that are likely to escalate the chances of its spread. Nevertheless, the practice of Eid-al-Adha among the Somali pastoralists enhances their exposure to the RVFV. During this occasion, animals are killed, and portions are given to the poor. During the 2006 and 2007 outbreak of RVF, a ban on this practice in northern Kenya supported by the local imams proved to be a critical step toward reducing human and animal morbidity and mortality caused by RVF alongside other measures, like a government-led ban on animal slaughtering, restrictions on movement of livestock, and implementation of vector-control programs.^24^

Other ritual events where the slaughter of animals takes place among the Somali pastoralists include dowry payment and wedding ceremonies.^1^ Should these occur during the outbreak of RVF, these ritual events may expose the people involved to a high risk of contracting RVF. During the 2006 and 2007 RVF outbreak in northeastern Kenya, family and community prayer meetings presided over by Muslim leaders (Sheikhs) were called. During this time, the Sheikhs recited the Quran to heal the sick who were suffering from RVF. Animals were slaughtered during the prayer meetings, and because this coincided with the epizootic, there was accelerated risk of transmission and spread of RVF, especially if the animals slaughtered were infected with RVFV or uninspected by health officials. The prayer meetings presided over by the Sheikhs to heal the sick reveal the worldview of the Muslims, which is dominated by the belief in Allah and its intricate interconnection with the Muslim life and health. The belief in Allah as the controller of life could lead to failure to recognize the real cause of a disease and by extension, the necessary control measures. Therefore, more scientific evidence is required to reveal the extent to which the belief system of Muslims influences their health-seeking behavior in remote contexts.

#### Food preparation and consumption practices.

According to Jost and others,^1^ the main benefits derived from livestock include meat, milk, ghee, and fat. Steers are raised for communal ceremonial feasts. Sheep and goats are kept for meat consumption or trading off to acquire cattle to improve herd structure.^31^ As such, milk and meat are part of pastoralists' diet. Fresh or curdled cow's milk, occasionally supplemented with steer's blood, forms 80% of their diet.^1^ This diet may pre-dispose the people to RVF in the event that the raw milk and blood is from an infected animal. There is a general perception among the pastoralists that raw milk and blood are more nutritious and hence, provide the needed energy for the youthful herders who move with the animals in search of pasture and water. Furthermore, fat in mutton is drained through boiling the meat, and the resultant fatty liquid is prepared for use in treating common ailments, such as ulcers and diarrhea. As a first line of treatment within the confines of home care, evidence suggests that the fat has also been used to treat patients manifesting RVF symptoms, such as fever and bloody diarrhea.

### Livestock production system.

#### Mobility of herds.

Traditionally, mobility of the herds is the basic requirement for pastoralism with a view to avoid overexploitation of pastures. There are daily and seasonal types of cattle mobility. Under daily mobility, the head of the household chooses a different grazing route after every 2 days according to the herd's needs. These daily movements involve small animals (mainly sheep and goats) and hardly exceed 5 km from the homestead.^30^ However, seasonal migration takes longer distances and durations of 4–5 months, and it is done by young men who move their cattle camps in the event of drought to where there is new pasture.

During seasonal movements, only lactating cows with newborn calves are left at home to provide the family with milk. Another form of movement among pastoralists is shifting of households.^30^ This is movement of the entire household, and it occurs at an interval of 5 or more years, mostly when severe drought strikes, leading to a shortage of water in the neighborhood. Mobility of the herd may be a pre-disposing factor to RVF by reason that, during movement, the herdsmen rely heavily on the animals and their products for food, mainly milk and blood. Because most of the range lands are far from animal healthcare services, surveillance for early detection of RVF in the mobile herd is a challenge to veterinary officials, a fact that may expose the herdsmen to RVF risk should they consume raw milk, blood, and even uninspected meat from an animal suffering from the disease. In northeastern Kenya, large livestock animals have to be moved during dry seasons in search of pasture and water, which leads them into different ecosystems and brings them into contact with wildlife and different vector communities.^48^ In the Ijara district, for example, livestock (cattle) is driven over long distances toward the Tana River delta or into the Boni forest. This mobility pattern into different ecosystems may result in transmission of RVF through the vectors. It has been suggested that cryptic existence and persistence of RVFV often without any manifestation of disease in man or animals have prevailed in many African countries, and there is potential for more serious epizootics and expansion, which must be seriously considered.^46^ Neutralizing antibodies to RVFV have been shown in wildlife in Kenya, including African buffalo, black rhino, lesser kudu, impala, African elephant, kongoni, and waterbuck. This raises the possibility that wildlife may be reservoirs for the virus during interepidemic periods and play a role in amplifying the virus during epizootics. As the human population continues to grow accompanied by increased livestock ownership, there is more pressure on available pasture for domestic animals and human settlement, with consequent invasion of wildlife territory in search of pasture and food. This may bring livestock and herders in contact with cycles of transmission of disease between wildlife and mosquitoes. Similarly, Aagaard-Hansen and others^54^ observed that nomads have differential exposure to diseases compared with settled populations, primarily because of their mobility, although Sheikh-Mohammed and Velema^55^ affirm that they may also avoid some health risks because of their movements.

#### Drought-related risk aversion strategies and RVF epidemiology: herd division, herd dispersion, and diversification strategies.

Over time, nomadic pastoralists have evolved a number of drought-coping strategies to reduce the risk of losing their livestock. These drought-tailored risk aversion strategies also have important bearings on their exposure to RVFV infection. In the same way that agricultural communities adopt practices, such as multicropping and maintenance of reserve granaries in areas of risky agricultural production, pastoral risk aversion strategies focus on herd modification actions, like diversification of species, dispersion, herds division and expansion, and migration.^25^,^29^ These mechanisms are likely to either prevent or expose pastoralists to the risk of infection with RVFV and should be subjected to scientific enquiry.

A common drought-related livestock management strategy adopted by pastoralists is herds division. Here, pastoralists split their herds into smaller groups to visit different grazing areas simultaneously. This has great potential for evading spread of RVF in the event of an outbreak from animal to animal. However, when sheep, which research has shown have the highest mortality and incidence rate during RVF outbreak, are grazed separately,^1^ the proximity of the high density of the sheep herd may escalate the transmission and spread of RVF to both the sheep and their herdsmen. It is not clear from the body of existing literature whether this aspect has been integrated into public education on RVF and management.

Herd dispersion is yet another drought-induced livestock size reduction risk minimization strategy practiced by nomadic pastoralists. This is where herds are regularly exchanged between herders to avoid the danger of losing the entire herd to drought, epidemics, or raids.^30^ Using recipients who live far away from each other facilitates this process, because regions are mostly affected differently by diseases, such as RVF. Pastoralists sustain this strategy by maintaining individual networks, where livestock transactions occur among people who are well-known to each other and share common vested interest in particular types of herds.^30^,^31^ In this respect, herd dispersion can help prevent the spread of RVF within the herd and hence, massive loss of livestock during epizootic.

Herd diversification is a practice by pastoralists where different types of livestock are kept to basically avert risks associated with disease and drought. This is because different species of livestock have different survival capacity in the face of calamities; hence, a farmer is able to spread the risk of losing the whole herd. On the herd diversification continuum, livestock owners' affinity with their livestock is quite a rational decision. The more arid an area is, the bigger the herd size to avoid the risk of starvation.^30^ Herd diversification makes more efficient land use possible, offers a broader spectrum of animal products, and secures a steadier supply of food. The different animals do not compete for pastures because of their different feeding requirements. Herds also vary in their susceptibility to disease, dry conditions, and theft.^30^

This explains why, under normal times, pastoralists are often reluctant to sell off their herds for fear of fetching low prices offered by traders, and this tendency persists to dry seasons. Some pastoralists retain their stock even in the face of severe droughts because of their own close attachment to their herds and to evade the likelihood of taking much time to rebuild their stock of herds when the situation changes. Thus, livestock sale is treated as a last option during drought. Although it is hypothetically tenable that livestock sales are inversely proportional to outbreak of RVF, there is no supportive empirical evidence to date suggesting, for example, that RVF outbreak stimulates massive livestock sales. Because herds differ in their susceptibility to RVF, with sheep being the most affected followed by goats,^1^ herd diversification may guarantee the security of stock by ensuring that the herd owner does not lose all of the livestock to RVF.

### The Institutional Environment.

#### Gender roles, pastoral labor organization, and exposure to RVF.

The issue of gender presents yet another fundamental aspect of social research on RVF. A major socioeconomic pattern in Africa is that pastoral women are subjected to a relatively inferior economic status, such as making decisions regarding milking, managing calves, goat kids, and lambs, and deriving their cash from sale of dairy products. In most pastoral societies, milking and management of milk resources are disproportionately done by women, except for instances where the cattle have to move to far distances. In sub-Saharan Africa, women frequently spend more time than their husbands in animal care.^26^ This led Dahl^27^ to conclude that pastoralism is a form of production in which the contributions of males and females are neatly interwoven. In the analogous terminologies of Jokes and Pointing,^26^ whereas women are associated with livestock as the means of subsistence as “milk managers,” men are associated with animals as wealth as “managers of herds.” This social disposition may expose women to risks of contracting RVF, because they get into contact with these animals on a day-to-day basis. Women do all of the work concerning animal products, like milking, slaughtering the small animals (goats and sheep), processing the milk, and caring for the hides and skins of slaughtered animals.

Among pastoral nomads and other herders in the arid regions of Africa, men and women are likely to be differentially exposed to RVF infection depending on the role specifications traditionally ascribed to them. For example, one study^4^ revealed that male participants were nearly three times more likely to be seropositive than female participants, a picture that was equally noted in the 1997 RVF outbreak investigations in northeastern Kenya.^7^ This is particularly so because men, particularly the herders, interact closely and for longer periods in isolation with animals during their seasonal movements in search of pasture. During this time, they are confronted with many risk factors, which increase their vulnerability to RVF. For instance, the reality of having to solely rely on raw cattle milk, blood, and uninspected meat during their seasonal movements confronts them with higher risk factors than women and herd owners. This finding is consistent with that of Aagaard-Hansen and others,^57^ who also found that gender roles cause differential exposure to diseases, particularly neglected tropical diseases (NTDs), such as trachoma and schistosomiasis.

However, in the study by LaBeaud and others,^4^ the difference in the seropositivity among males and females was not explained on the basis of reported animal or non-animal exposures, which were comparable and not statistically different between genders. Instead, LaBeaud and others^4^ concluded that increased seropositivity among males was attributable to biological factors on the ground and that the outcome of infection and resultant immune response to other viruses have been linked to gender differences.^4^ This remark points to the conclusion that it is not clear how role differentiation among female and males among nomadic pastoralists influences detection, spread, and prevention of RVF.

The unclear nexus between RVF outbreak and gender of persons infected with the virus commonly reported in studies is attributable to the nature of labor organization of the pastoral communities and inadequate recognition of the unique contributions of the women to livestock production activities, healthcare, and treatments. One reason that the aspect of gender has not been visible in the analyses of pastoralists health is the overtly neglected role that women play in livestock management. Although women play a crucial role in disease control because of their being at the center of milking, which enables them to detect signs of illness in livestock, like sudden drop in the milk yield, this is seldom recognized in the diseases research and management programs.^28^

In Chad and Uganda, women themselves look for the roots and leaves needed for treating the animals, and even men call on the knowledge of their wives on this matter.^28^ The literature linking this important role of women in pastoral communities to detection, prevention, and control of RVF is still scarce.

Furthermore, nomadic pastoral engagements are highly labor-intensive. Work is carried out almost entirely by household members, and each member of the household has a special task relating to animal care according to age and sex. From the age of 4 years old, both boys and girls are trained to look after the cattle and goat kids. Later, their tasks are divided into women's and men's work. This implies that almost the entire population is exposed to infection with RVF at a young age, but most of the past studies and actions on and about RVF, including public education and RVF prevention campaigns, have disproportionately focused on adults, particularly the herd owners, most of whom do not move with the animals.

#### Indigenous knowledge base of RVF among the Somali pastoralists.

Scholars have noted that few studies have been mounted to establish the local communities' knowledge of RVF in epidemic-prone areas. Nevertheless, it is worth appreciating that using the indigenous knowledge of a community is a viable undertaking, because it has the potential to support disease surveillance, early warning systems, and prevention measures, thereby substantially reducing the risk of massive infections and loss.^32^ Similarly, Martin and others^49^ insist that, although for millennia, people have used homegrown veterinary skills and techniques to keep their animals healthy, it is only in the last decade that people's local knowledge and skills received much scientific attention under the rubric of ethnoveterinary medicine.

Nyamanga and others^50^ found out that farmers seek both curative and preventive medical services for their animals from the broad range of healthcare providers available to them within a pluralistic medical system. This calls for the integration of a pluralistic perspective into the planning and implementation of animal healthcare interventions and services. Furthermore, decisions regarding healthcare choices for livestock are based on perceptions of the cause of the health problem, belief in the efficacy of a given approach, and cost implications, particularly in resource-poor households, all of which need to be understood.^51^–^53^

In a study conducted by Jost and others,^1^ Somali pastoralists of northeastern Kenya proved to be adept at recognizing symptoms of RVF and risk factors, such as heavy rainfall and mosquito swarms. Sandik, which means bloody nose, was used by Somalis to denote disease consistent with RVF. They reported that sandik was previously seen in 1997 and 1998, the period of the last RVF epidemic. The pastoralists reported that high proportions of their goats, sheep, and cattle had been sick during the 12-month period from July of 2006 to June of 2007, which included the duration of the RVF outbreak.

In the same study, the highest morbidity rate was reported for goats, with 77% of goats in Garissa and Ijara districts reported to have fallen sick during this period. Although the frequency of diseases that pastoralists reported varied, RVF featured prominently for cattle, sheep, and goats. Four of the most commonly mentioned diseases for all four species as reported by Somali pastoralists in Kenya included tick-borne disease (geesdoor), RVF (sandik), lumpy skin disease (fuuruk), and foot and mouth disease (cacbeeb or habeeb). The Somalis consistently listed symptoms, such as abortion and froth emanating from the nose, as being indicative of a disease that they named sandik, and they associated this disease with heavy rain and mosquito swarms.^1^ Other symptoms included bloody diarrhea, coughing, salivation, pruritus, fever, and lachrymation.

The study by Jost and others^1^ revealed that sheep were most affected by the RVF outbreak: this species had the highest outbreak incidence, fatality, and mortality rates. Jost and others^1^ estimated that 88.3% of their sheep died during the outbreak compared with 56.2% of goats and 36.5% of cattle. Abortion rates experienced during the outbreak were high in the northeastern province of Kenya, where pastoralists estimated that 47.1% of pregnant cattle, 69% of pregnant sheep, and 62% of pregnant goats aborted because of RVF. Furthermore, the Somali pastoralists considered that RVF was the disease that had the highest impact on livestock-derived livelihoods for all four livestock species.

Timelines constructed in the villages based on pastoralists' recall of key events during the RVF outbreaks showed that the mean interval between the start of heavy rains and the first appearance of mosquito swarms was estimated to be 23.6 days. The mean interval between first appearance of mosquito swarms and first suspected RVF case in livestock was estimated to be 16.8 days.

The study by Jost and others^1^ points out the important role that indigenous knowledge of livestock keepers can play in veterinary surveillance.^33^,^34^ This critical aspect was ignored during the 2006/2007 outbreak, although results showed that the pastoralists, especially the Somalis, were aware of the unusually heavy nature of the rains and flooding before the outbreak of RVF in their areas. They also noticed mosquito swarms that were unusual because of their intensity and the physical characteristics of the species involved (*Aedes* spp.), and they noted unusually high morbidity and mortality in their herds, consistent with RVF.^5^,^7^,^35^ These facts were common knowledge among livestock owners well in advance of the detection of RVF by veterinary service surveillance systems.^8^ This suggests that veterinary surveillance systems could detect RVF earlier by taking advantage of livestock owner observations and indigenous knowledge through the integration of active syndromic surveillance, such as participatory disease surveillance (PDS), geared to the level of outbreak probability.^1^,^33^

## Sociocultural and Economic Dimensions of RVF: Economic Dimensions

The economic dimensions capture the role of institutions within the value chain and their capacity to enforce the rules to minimize health risks. They also reflect the magnitude of human and financial resources deployed in RVF prevention and management, the set of constraints and opportunities for resource mobilization encountered primarily by the relevant public institutions, and the range of effects that the outbreak of RVF has had on the livelihoods of the affected people.

### Response by national governments to RVF disease outbreak.

The influence of economic factors on RVF outbreaks has been documented, for example, in the outbreak that occurred for the first time in 2000 and 2001 in Yemen and Saudi Arabia.^47^ It was reported that the importation and smuggling of livestock from Somalia for the Eid-Al Kabeer celebrations during periods of high vector densities led to the outbreak in these Middle East countries.^47^ As a result, an array of measures have been mounted by various actors and institutions within and without the livestock food chain in an attempt to reduce risks in different countries.

The most recent outbreak of RVF occurred in Kenya in 2006/2007. In response, the Kenyan Government through the Ministries of Health and Veterinary Services deployed staff and resources to northeastern Kenya to contain the epizootic. However, according to Jost and others,^1^ veterinary personnel in northeastern Kenya were unable to mount an immediate response against the disease because of varied challenges. Reasons given for this were that most of the roads were impassable because of floods, and there was lack of suitable equipment, especially vehicles, insufficient personnel, and lack of funds.^1^ Key informants reported that the Ministry of Health responded when human cases started occurring.^1^ This team vaccinated livestock, treated sick animals that had other infections, provided insecticides, and took samples from suspected livestock cases. Human cases were also identified, managed, and sent for laboratory confirmation, and vectors were sampled and screened. Control measures used included closing livestock markets and butcheries, imposing movement controls and quarantines, and providing advice and warnings against drinking raw milk, slaughtering animals, or eating uninspected meat.

The study by Jost and others^1^ also highlighted weaknesses in both RVF preparedness and response. Late detection of RVF in both animals and humans meant that the disease was well-established in the livestock population before veterinary and public health interventions were initiated. Key informants reported the intentions to vaccinate areas surrounding infected areas in an attempt to control the spread of the disease. However, the disease was already widespread and present in the areas where vaccination campaigns were implemented by the time that the vaccination logistics could be coordinated. In part, veterinary services were limited by flooding and access to transport, and where they were available, their vehicles were in a poor state of repair; vaccine was often delivered by government health officials, who were targeting high-risks areas for human cases.

Early warning indicators and early warning processes need to be reassessed. The study by Jost and others^1^ highlights the importance of improved RVF preparedness and early warning systems. To be effective, early warning systems must provide information before the onset of events in a manner that allows authorities sufficient lead time to respond. Findings indicate that the observations by local communities of climatic, entomologic, and clinical events consistent with RVF within the known risk-prone areas were more timely and definitive risk indicators than the global early warning systems in place at the time of the 2006/2007 outbreak.

Furthermore, Jost and others^1^ hold that the use of vaccine in the emergency prevention and control of RVF outbreaks should be reconsidered. The Smithburn vaccine (Kenya Veterinary Vaccines Production Institute, Nairobi, Kenya) provides effective immunity against RVF after a single inoculation, making it an appropriate choice for emergency vaccination programs, although it does cause abortions in sheep, and the vaccine virus can be transmitted by vectors. It is postulated that vaccination in the 2006/2007 RVF outbreak was probably not effective because of the constraints to timely delivery of vaccination as part of the response plan linked to early warnings. It is likely that routine preventive vaccination would be epidemiologically more effective than heroic attempts to deliver emergency vaccination in response to early warnings, but this probably does not make economic sense given the infrequency of outbreaks in the region. One sustainable solution would be the development of multivalent vaccines (vaccines that combine valencies that treat more than one disease) that justify more frequent vaccination with an RVF component. Consideration should also be made for a phased response that minimizes the risk of incorrect decisions and maximizes preparedness in the event of an outbreak.^34^ Initiatives, such as the risk-based decision support tool, can be further enhanced by continued research.

### Resource base and institutional capacity for RVF management in Kenya.

Continued monitoring and reporting of cases has been widely recognized as a vital step toward combating the devastating health and economic ramifications of an RVF outbreak. Also, enhanced surveillance has been recommended as a necessary measure.^40^ Accordingly, since the 2006/2007 outbreak of RVF, the Department of Veterinary Services (DVS) of the Kenyan Ministry of Livestock Development has been conducting continuous vaccination and education campaigns. For instance, clinical treatments were done in parts of Danyere Division of Garissa County at the close of 2009 for the purpose of contributing to disaster preparedness through extension of predicted disease outbreaks and enhancing livestock health and production. Of the total of 116,026 livestock species vaccinated, 36,389 were vaccinated against RVF.^41^

Some influential studies have pointed out that predictive epidemiological inputs can drive prophylactic vaccination campaigns in the high-risk areas provided that there is a sound economic justification and the corresponding institutional capacity exists.^7^,^23^ However, in the Kenyan context, this has been difficult to achieve. For example, the capacity of the DVS to effectively respond to an outbreak of RVF or its signs of possible outbreak through vaccination has been hampered by several scenarios. There is a lack of adequate equipment, such as protective clothing, animal counters and markers, drenching guns and cannulas, and camping gear.^41^

Another common major challenge encountered is communication breakdown between the field team and the coordination team at the Garissa Veterinary Office because of wide spatial distances and flooded roads during heavy rains. Thus, these interventions fail to be satisfactory because of the untimely delivery of vaccines and shortage of drugs or ice. Consequently, communities lose confidence in the initiatives, with a resultant drop in vaccination uptake.

A combination of active and passive surveillance between animals and humans is also a distinct strategy for preventing RVF outbreak and diluting its economic impact. Passive surveillance is concerned with reports on upsurges in abortions, hemorrhagic syndromes, and deaths, especially in young animals. Under active surveillance, the DVS's personnel are required to be on the alert by intensifying disease search for RVF disease with emphasis on areas that experienced the disease in both humans and animals during the last outbreak of 2006/2007. In both cases, a participatory disease search approach, in which pastoralists prioritize diseases and conditions against livestock species as they occur at all times, is followed.^41^

### Economic effects.

Other than human illness, disability, and suffering, outbreaks of RVF can result in devastating economic losses at household and national levels. This arises from the vast body of evidence provided by both anthropological and ecological studies confirming that pastoralists usually incur great losses, including reduction in milk production and deterioration of animal health, when unusually heavy rainfall resulting in massive floods occurs.^36^ It has been noted that livestock producers are negatively affected by measures that bar exports or slaughter and remove or reduce opportunities for earning income.^37^ For example, an analysis of the public health burden of RVF outbreaks measured in disability-adjusted live years (DALYs)^38^ indicated that the 2006–2007 outbreak resulted in 3.4 DALYs per 1,000 people and household costs of about Ksh 10,000 (equivalent to US$118) for every human case reported; the cost of the outbreak to the Kenyan economy was estimated at US$30 million.^38^ During the 1997/1998 RVF outbreak in Kenya, livestock owners reported losses of approximately 70% of their sheep and goats and 20–30% of their cattle and camels.

Similarly, a 2007 rapid assessment of the effects of short rains conducted shortly after the RVF outbreak of 2006–2007 indicated that, in Garissa, RVF had claimed lives of hundreds of goats and a substantial number of cattle, with some herders reported to have lost up to 75% of their goat populations.^39^ The results are as presented in Table 2.

Accordingly, the situation as described in the report succinctly captures the economic implications of an RVF outbreak. In all of the areas assessed, there was a similar trend of reduced livestock prices compared with prices before the onset of rains. Although reductions in prices are necessitated by the usual factors of demand and supply, the sharp reductions in the prices were occasioned by two compounding issues that were outside the domain of the demand and supply curve, namely outbreak of the RVF and closure of all of the feeder markets and the central market in the Municipality of Garissa.^39^

The report^39^ further noted that the outbreak of RVF in some areas of the district (Garissa) prompted the quarantine closure of livestock markets in all of the centers in the district. This disaster occurred at a time when the pastoral communities received abundant rain, and with the plenty of pasture, their livestock were fattened and in good shape. Having received abundant supplies of both pasture and water—the two key parameters used to measure the agronomical wellbeing of the pastoralists—the RVF came with huge losses of livestock followed by quarantine and the closure of the markets. This sorry state of affairs constitutes what experts on pastoral policies refer to as the painful paradox of the rich–poor.^1^ This anomaly is explained as the herders having their stocks fattened and ready to fetch a good price in the market, but the markets are closed; therefore, they cannot benefit from their perceived wellbeing. This phenomenon has crippled the pastoral economy and increased the levels of destitution to alarmingly high levels. The inability of the herder to sell his surplus of stock and pay back the debt that he accrued during the dry spell forces him to continuously borrow, a condition economists call debt coupling.^1^ This economic limbo has negatively affected the social relations of the debtors and creditors. The two pinnacles of the pastoral economy, in some cases, resulted in the fighting between these two groups. Furthermore, the ultimate costs of the 2006–2007 RVF outbreak to the livestock industry were estimated to be Ksh 4 billion.^39^

## Conclusion

The literature review provides detailed analysis of the sociocultural and economic dimensions of RVF. Although this work manages to consolidate the sociocultural and economic constructs of RVF disease, it identifies the paucity of social research on RVF .The review found that most of the studies conducted on RVF were, by and large, veterinary- and health-oriented, with very little focus given to the sociocultural and economic aspects. Collaboration between epidemiologists, veterinary and medical health officials, and social scientists is increasingly needed. It is our belief that cross-disciplinary research is likely to increase our understanding of all of the dimensions of RVF and thereby, inform the design of comprehensive and cost-effective measures for prediction, detection, and response to RVF. In particular, it may provide a basis for establishing comprehensive RVF outbreak preparedness and inform advocacy dialogues as well public education campaigns. The review strongly suggests that timely outbreak response requires effective early warning and surveillance systems as well as public education. These have the potential to reduce human illnesses and deaths associated with RVF outbreaks. Relevant and cost-effective interventions can also be secured if livestock value chain analysis is undertaken together with risk analysis to determine the stages at which pathogens are maintained and transmitted. This kind of analysis will help in understanding people's behaviors and motivations within the value chain, especially during epizootics, for identification of appropriate people-centered interventions. Finally, it is acknowledged that gender dynamics have important ramifications for RVF infections, but the manner in which male–female disaggregated roles among pastoralist communities differentially expose women and men to infection and spread of RVFV is not yet fully understood. This calls for more research on this front.

## Figures and Tables

**Table 1:** Literature search returns and numbers of materials reviewed

Search terms	Return	Materials selected	Number
“Rift Valley Fever”	1,321	Peer-reviewed articles	33
“Rift Valley Fever in Kenya”	117		
“Social determinants of Rift Valley Fever”	0	−	−
“Sociocultural factors of Rift Valley Fever”	0	−	−
		Unpublished reports and papers	7
		Total materials selected	40

**Table 2:** Changes in the livestock prices at the Garissa Municipality Market (February of 2007)

Herd	Before the rains (Ksh)	After the rains (Ksh)
Camel	20,000–32,000	15,000–23,000
Cattle	12,000–18,000	6,000–10,000
Goats	1,600–2,200	1,000–1,300
Sheep	1,000–1,400	600–900

Source: DVS, unpublished data. $1 = 85 Ksh.
